# Editorial: Imaging assessment of response to immunotherapy

**DOI:** 10.3389/fonc.2022.1116315

**Published:** 2023-01-20

**Authors:** Mariaelena Occhipinti, Andrea R. Filippi, Sean Walsh, Jan P. van Meerbeeck

**Affiliations:** ^1^ Medical Department Radiomics (Oncoradiomics SA), Liege, Belgium; ^2^ Department of Clinical, Surgical, Diagnostic and Pediatric Sciences, University of Pavia, Pavia, Italy; ^3^ Division of Radiation Oncology, Fondazione Istituto di Ricovero e Cura a Carattere Scientifico IRCCS Policlinico San Matteo, Pavia, Italy; ^4^ Department of Pulmonology & Thoracic Oncology, Antwerp University Hospital, Edegem, Belgium

**Keywords:** immunotherapy, imaging, radiomics, response to therapy, cancer

In the last few years, immunotherapy has emerged as the therapeutic option of choice for several different advanced diseases, especially in the oncology field. Many drugs have received regulatory clearance both in the EU and US for melanoma, lung, bladder, renal and oropharyngeal cancers among others. In turn, the tools to asses therapy efficacy and response have evolved to better understand the disease progression and the effects of these new therapies. This is the case for example of the RECIST criteria, developed originally for chemotherapy, that was updated with immune-related response criteria (irRC), immune-related RECIST (irRECIST), and immune RECIST (iRECIST). The review paper of Berz et al. sheds some light on the current landscape of imaging assessment criteria for immunotherapy. The reliance on the subjective assessment of the radiologist subjective has been seen in the past as one of the main limitations, and the trustworthiness of the response prediction is deeply linked to the experience of the reader and their personal judgment. Efforts to reduce and minimize these effects have been various and start from simple but more structured approaches to tumor characteristics. The paper of Belkouchi et al. reports the use of tumor load (i.e. tumor volume) to predict Overall Survival (OS) and Progression Free Survival (PFS), proving that adding this parameter to more traditional RECIST scoring, improved sensibly the prediction performance and the patients stratification. They also underlined the importance of early-stage response in the long-term outcome of the therapy as also reported by Ma et al., for melanoma patients treated with combination Ipilimumab/Nivolumab. Imaging assessment can be also instrumental in preclinical and animal studies, to better understand the possible characteristics of the response and the landscape of efficacy of candidate drugs, active towards specific molecular pathways. The combination of advanced PET/CT imaging, using a specific radiotracer for vascular adhesion protein-1 (VAP-1), has been exploited by Viitanen et al. to monitor pharmacodynamic effects of a novel FAP-IL2v immunocytokine in melanoma mice. The *in vivo* PET/CT imaging has been complemented with *ex vivo* autoradiography and histological analysis with immunofluorescent staining. The findings allowed the authors to elucidate the pharmacodynamic effect of the drug and to suggest the novel radiotracer as a possible imaging agent *in vivo* to follow therapy response at a lesion level.

Trying to go beyond the current limitations of imaging assessment of therapy response, in the last years Artificial Intelligence and in particular, radiomics, have emerged as innovative approaches for several different diagnostic and prognostic tasks. The use of advanced medical image analysis is gaining considerable traction also in cancer immunotherapy. The most interesting possibility is to predict from baseline or early follow-up scans, the overall response to therapy. Radiomics could help in the stratification of patients based on response profiles and characteristics or identify patients’ imaging phenotype which could benefit from one therapeutic approach more than others. Gabrys et al. reported for example the prediction of hyper progression in metastatic melanoma, both at the lesion and patient level, based on radiomics analysis of PET/CT scans. As reported by Tong et al. the combination of radiomics and clinical features can be exploited to predict the tumor immune profiles of NSCLC lesions, which in turn is linked to the lesion response to immunotherapy. This can help stratify patients who will more likely benefit from therapy and spare burdensome treatments and hospital resources. However, the road to routine clinical application of these radiomics strategies is still long and there are several roadblocks that need to be surpassed to allow for the real integration and acceptance of radiomics, and Artificial Intelligence in general, as ancillary techniques for the clinical decision support system. One of the main issues is the lack of mutual understanding and exchange between researchers and developers and clinical experts who, more often than not, speak two different languages. Sharing a common understanding of the “what”, “who”, “how”, and “why” is the first fundamental cornerstone of this transition. Also, the lack of prospective clinical trial validation and real-world data on their usage and effectiveness is seen by many as one of the strongest shortcomings. Notwithstanding these limitations, the implementation of automatic or semi-automatic image analysis methods to assess response to immunotherapy could represent the turning point in the transition towards a more patient centric approach to cancer care.

## Author contributions

All authors listed have made a substantial, direct, and intellectual contribution to the work and approved it for publication.

